# Current State and Challenges of Natural Fibre-Reinforced Polymer Composites as Feeder in FDM-Based 3D Printing

**DOI:** 10.3390/polym13142289

**Published:** 2021-07-13

**Authors:** Nishata Royan Rajendran Royan, Jie Sheng Leong, Wai Nam Chan, Jie Ren Tan, Zainon Sharmila Binti Shamsuddin

**Affiliations:** School of Engineering, UOW Malaysia KDU University College, Jalan Kontraktor U1/14, Seksyen U1, Glenpark U1, Shah Alam 40150, Selangor, Malaysia; 0118234@kdu-online.com (J.S.L.); 0118586@kdu-online.com (W.N.C.); 0118863@kdu-online.com (J.R.T.); zainon.s@kdu.edu.my (Z.S.B.S.)

**Keywords:** natural fibre, additive manufacturing, filament, surface treatments, optimisation, printing parameters, mechanical properties

## Abstract

As one of the fastest-growing additive manufacturing (AM) technologies, fused deposition modelling (FDM) shows great potential in printing natural fibre-reinforced composites (NFRC). However, several challenges, such as low mechanical properties and difficulty in printing, need to be overcome. Therefore, the effort to improve the NFRC for use in AM has been accelerating in recent years. This review attempts to summarise the current approaches of using NFRC as a feeder for AM. The effects of fibre treatments, composite preparation methods and addition of compatibilizer agents were analysed and discussed. Additionally, current methods of producing feeders from NFRCs were reviewed and discussed. Mechanical property of printed part was also dependent on the printing parameters, and thus the effects of printing temperature, layer height, infill and raster angle were discussed, and the best parameters reported by other researchers were identified. Following that, an overview of the mechanical properties of these composites as reported by various researchers was provided. Next, the use of optimisation techniques for NFRCs was discussed and analysed. Lastly, the review provided a critical discussion on the overall topic, identified all research gaps present in the use of NFRC for AM processes, and to overcome future challenges.

## 1. Introduction

In recent years, the scientific and industrial communities have begun to acknowledge the need for environmentally friendly processes, materials and waste management. As environmental awareness is on the rise, product designers and engineers recognise the impacts of their decisions to use non-renewable resources or non-biodegradable resources. The trend in the industry has begun to shift from using conventional plastics, which are mostly non-biodegradable, to using bio-composites which depend on the polymer matrix that can be biodegradable or carbon neutral. This is primarily due to the great impact of synthetic polymers towards the environment. As part of the efforts to increase the recycling of plastics and reduce its production, researchers have developed natural fibre-reinforced composites (NFRC). These composites are polymers mixed with natural fibres, such as hemp, jute, rice stalk, kenaf and rice husk. The result is a material that has far superior mechanical properties and reduced cost, depending on the type of polymer matrix or even biodegradable [1]. NFRCs are gaining popularity mainly due to their high strength to weight ratio, and thus they can be 25% to 30% stronger than glass fibre composites of the same weight [2]. Because of its various benefits, the global use of NFRCs is starting to grow rapidly. In recent reports, the NFRC market value in 2016 was 4.46 billion USD and is forecasted to grow with a compound annual growth rate of 11.8% from 2016 to 2024 [2]. The use of NFRCs is mainly to produce housing decks and railings but has recently gained popularity in the automotive sectors with manufacturers producing car interiors from NFRCs. Current processing techniques for NFRCs do not differ much from the processing of conventional plastics. Techniques, such as extrusion, compression moulding and injection moulding are used to process and form NFRCs just as they would be used for conventional plastics [3]. As mankind steps into the fourth industrial revolution, the focus has shifted to interconnectivity, automation, machine learning and real-time data. The digitalisation of the manufacturing industries is rising rapidly [4]. There has been a rise in the need for rapid prototyping and ability to produce customised parts quickly and at a low cost. Conventional methods of manufacturing remove material from a large piece of stock, which is known as subtractive manufacturing. These processes are sometimes complicated and time-consuming for complex parts as many parameters need to be considered, such as tool speed, draft angles and tooling types. Even with computer numerical control (CNC) technology, the cost of producing a one-off part with CNC is much higher than using additive manufacturing (AM) techniques. Therefore, researchers have identified AM as one of the more essential components in the rise of the fourth industrial revolution [5]. Almost all of the AM technologies operate based on the same principle, whereby a part is produced through the deposition of material layer by layer. The part starts as a computer-aided design (CAD) model, which is then loaded into the special software known as a slicer. The slicer slices the CAD model into the desired layer height and produces the code to control the printer for each layer. Currently, AM technology is more widely used for polymers. However, although rare, there are commercially available machines that can perform additive manufacturing on metal [6]. As AM technology gains popularity amongst researchers and the industrial sector, there have been efforts to utilise NFRCs in AM. One of the most predominant and popular techniques for AM is fused deposition modelling or sometimes known as fused filament fabrication [7], whereby a polymer filament is extruded through a heated nozzle while the printer traces out the cross-section of each layer.

The use of NFRCs in AM is still a relatively unexplored field as literature on this topic is limited. However, the ability to use NFRCs in AM proves to be beneficial as it can greatly speed up the prototyping process when working with NFRCs. As compared to conventional processes like the injection moulding, a mould needs to be produced before the prototype can be tested. These processes are costly and limit the number of prototypes that can be produced. If NFRCs can be used in AM reliably, the prototyping process can be significantly hasten and at a much lower cost. Furthermore, because there is no need for a mould, more iterations of a product can be tested.

Therefore, the target of this review is to provide a background of the current state of using NFRCs in AM. The review aims to provide an insight into how NFRCs are currently used for AM. The parameters of all stages, involving from raw materials to the final printing process are discussed to provide readers with an insight into what is involved and what affects the properties of a NFRC for use in AM. First, an overview of the currently available AM technologies and works which utilise NFRCs in AM is provided. Next, preparation methods and parameters involved in producing the NFRCs are discussed, followed by the process of producing the filament for use in AM. Printing parameters involved and how they affect the mechanical performance of the composite are discussed in the following sections. Finally, the mechanical properties reported by various researchers who use NFRCs in AM are discussed, followed by an overview of how optimisation methods are employed to optimise the mechanical properties of these composites.

## 2. Additive Manufacturing Technologies

Additive manufacturing, commonly referred to as 3D printing is the process of fabricating three-dimensional solid objects from a digital machine. The object is typically created by depositing materials layer by layer until the overall object is fabricated successfully. 3D printing is getting more popular as compared to conventional machining techniques, such as milling, drilling, cutting, etc. AM technology is superior to conventional techniques when the object complexity increases. A few technologies that are generally used in 3D printing include fused deposition modelling (FDM), whereby a polymer material is extruded layer by layer to form a 3D object. In stereolithography (SLA), a photosensitive resin is hardened by a UV projector layer by layer. Meanwhile, in selective laser sintering (SLS), a laser sinters polymer or metal powder on a powder bed and forms each layer. Direct write (DW), which is very similar to FDM but does not involve material heating, is directly extruded. This technique is usually used for clay or concrete. Lastly, binder jetting (BJ) is very similar to SLS but instead of laser sintering a binder is injected onto a powder bed to form each layer [8]. FDM-based 3D printing is a popular additive manufacturing technology that is widely used, with thermoplastic materials which have a melting point of lower than 300 °C. For the printing process, the feeder, which is commonly known as a filament, is fed into the 3D printer, whereby the polymer filament is melted and extruded from a temperature-controlled nozzle, also known as the extrusion head, onto a build platform. Depending on the type of polymer, the build platform can be heated or left as it is. The object is created by the layering of thermoplastic at a certain degree of accuracy, whereby either the nozzle or build platform moves in the x-axis, y-axis and z-axis. Type of thermoplastics material that works in the process of FDM includes acrylonitrile butadiene styrene (ABS), polylactide acid (PLA) and high-density polyethylene (HDPE).

**Figure 1:**
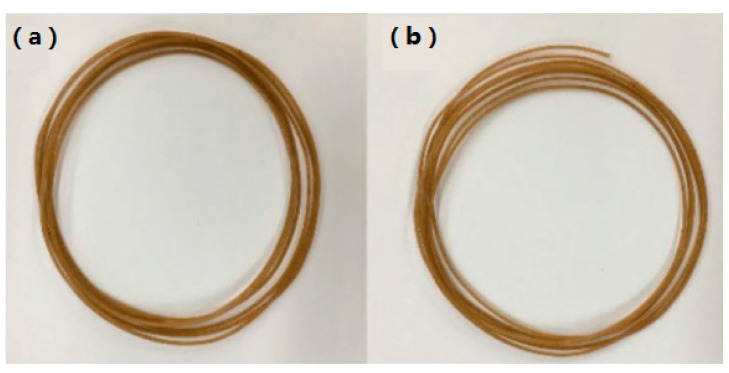
PLA/teak wood flour (TWF) composite filament of (**a**) 74-µm TWF (**b**) 125-µm TWF [9] Licensed under Creative Commons Attribution 4.0 International License.

**Figure 2:**
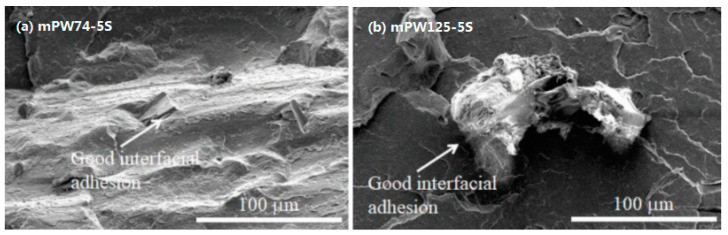
SEM micrograph of modified PLA/TWF composite filament of (**a**) 74-µm TWF (**b**) 125-µm TWF [9] Licensed under Creative Commons Attribution 4.0 International License.

However, there are few significant drawbacks of FDM as compared to stereolithography (SLA) and selective laser sintering (SLS), as well as similar standard processing methods (e.g., injection moulding). The quality and mechanical properties of the printed product are not as good, mainly due to the layering technique in printing which adds more points of contact/failure, and the inevitable presence of voids. Furthermore, the mechanical properties of 3D-printed objects are anisotropic and highly dependent on the processing parameters [8]. On the other hand, the small nozzle size of this technology could have a drawback on printing composites like NFRCs because the fibres exist as small particles in a polymer matrix. Moreover, it is not always possible to produce a homogenous mix of polymer and natural fibre matrices, whereby agglomeration might occur in the filament which will clog the nozzle. This phenomenon is shown in Petchwattana [9] studies, in which a modified PLA/Teak wood flour composite filament was produced and the specimen with 125-µm particles clogged the printer nozzle. Figure 1 shows the composite filament for both 74 µm and 125 µm teak wood flour filler. The SEM micrograph of the modified PLA/Teak wood flour composite filament is shown in Figure 2, whereby good interfacial adhesion can be seen. Nevertheless, due to the large particle size the specimen with 125-µm particles still clogged the printer nozzle.

### 2.1. Thermoplastics for Additive Manufacturing

Thermoplastics polymers are plastics that return to the solid state upon cooling after being moulded above the threshold temperature [10]. In terms of choosing a suitable polymeric matrix material, thermoplastics are the most compelling choice. This is due to its various advantages, such as low moisture absorption, mechanical properties and infinite shelf life [11], low cost [12], better design flexibility and simple processing technique [13]. Moreover, thermoplastics tend to stay attached during the 3D-printing process [14]. Polymeric matrices can be categorised into non-biodegradable and biodegradable polymers. A variety of standard thermoplastics that are suitable and generally referred to as ‘plastics’ include acrylonitrile butadiene styrene (ABS), polylactide acid (PLA), polypropylene (PP) and polyethylene (PE) [15]. However, in terms of additive manufacturing, only polymers with working temperatures of below 300 °C are suitable, as FDM printing temperature is around 300 °C [16]. The comparison of melting temperatures of some common thermoplastics is shown in Table 1. As seen in Table 1, all common thermoplastics have a melting temperatures below 300 °C, which indicate the suitability of these thermoplastics to be utilised in FDM 3D printing.

ABS polymer is considered as one of the most common materials that are widely used as the filament in AM [8]. Nowadays, ABS-made applications in various industries can be found easily, with their wide range of manufacturing methods, including injection moulding and filament for 3D printing [22]. It comprises the combination of acrylonite, butadiene and styrene monomers to form a single polymer with great mechanical performance [15], durability as well as ease to print [8]. With stresses above their tensile strength, ABS has strong durability, but elasticity modulus and hardness are higher. Meanwhile, PLA is one of the most popular thermoplastic polymers because it is inexpensive with high modulus and strength [23]. Its low melting point requires lesser energy in 3D printing [24]. Its renewability and biodegradability have made it popularly used in a wide range of applications in the industry, especially in additive manufacturing. Besides, PLA does not emit any unwanted gases or unpleasant smells during the process [25]. Melnikova et al. [26] had successfully produced a flexible cloth with PLA by using FDM technology. PLA had also been added with various compatibilizers or reinforcing agents to mainly enhance its properties [27].

In another case, high-density polyethylene (HDPE) poses excellent mechanical properties, such as high stiffness and melting point, as well as high compressive tensile strength that outperforms other polyethylenes (e.g., low density polyethylene) [15,28,29]. It has a high strength-to-density ratio as compared to another thermoplastic. With its advantages, such as watertight, lightweight yet strong, recyclable and FDA approved, it has become one of the ubiquitous plastics used in different applications and commercial products. Schirmeister et al. [30] reported the success of printing HDPE by FDM technology with similar mechanical properties as compared to the injection-moulded HDPE, without significant warpage and void formation. HDPE is also a reusable thermoplastic polymer, unlike thermoset polymers [31]. Moreover, HDPE is easy to transform into new material through recycling. Recycling plastics could cause a significant impact on the environment, leading to more sustainability. Regardless, a recycled high-density polyethylene (rHDPE) still possess its unique characteristics even as a recycled material, such as chemical resistance, moderate tensile and impact strength, as well as excellent abrasion-resistant properties [32]. Even though research on rHDPE for additive manufacturing is scarce, the availability of HDPE waste had encouraged efforts to use recycled materials for more beneficial use, such that recycled HDPE from milk jugs were adapted to 3D printing in various recycling projects [31]. Baechler et al. [33] also reported the success of using the extruded recycled HDPE polymer to produce increasing successful parts with the 3D printer. Moreover, Angatkina [31] reported that rHDPE is suitable for 3D printing without any significant differences in mechanical properties as compared to the pure HDPE polymer.

### 2.2. Natural Fibre-Reinforced Composites in Additive Manufacturing

Natural fibres (NF) are known as hair-like raw material that is obtained from animals, plants or mineral sources. The main difference between animal and plant fibre is that animal fibre is mainly made out of protein, whereas plant fibres are composed of cellulose. Examples of natural fibre that is derived from plants include rice husk, wheat, hemp, bamboo and cotton. Thiranan Kunanopparat et al. [34] reported that the mechanical properties of wheat composites were less sensitive to thermal treatment when the fibre content was increased. The processing temperature was reported to be an important parameter that affected the physical adhesion, whereby this physical adhesion played a big role in the mechanical properties of the composites. Chen et al. [35] reported that bamboo fibres have much lower volumes of fracture behaviours, high proportions of fibre dissociations and matrix failure due to its hierarchical fibrous woven structure. On the other hand, Sueli Aparecida de Oliveira et al. [36] reported that composites that have natural cotton fibres as fillers exhibited a reduced environmental impact as compared exclusively to PLA or PLA/thermoplastic starch (TPS)-based thermoplastics. Moreover, the natural cotton fibre composites had a better overall performance, under the eco-efficiency perspective. V. Mazzanti et al. [37] reported that the presence of even small amounts of hemp fibres accelerates PLA degradation to an extent similar to that of residual water in non-dried pure PLA. A natural fibre that can be found abundantly and replenished in a predictable period is known as renewable natural fibre. This NF is commonly used as a low-cost alternative to synthetic fibres (e.g., nylon) mainly due to its biodegradability, cost-effectiveness, satisfactory mechanical properties and low density [38]. Natural fibre generally has low densities, ranging between 1.1 g/cm^3^ and 1.6 g/cm^3^, and thus result in low weight [8]. Plant fibre generally contains 60–80% cellulose, 5–20% hemicellulose, while the rest constitutes lignin, waxes, moisture (up to 20%) [39]. Besides, the cellulose content or cellulose crystallinity is also a factor to be considered when choosing a reinforcement for polymer. For example, bast fibre with 50–90% cellulose crystallinity is utilised in various automotive components to achieve better mechanical properties of products in terms of modulus, strength and stiffness [40]. Moreover, rice husk natural fibre was introduced recently as reinforcement in polymer to form NFRCs. Their high cellulose content would result in higher lignocelluloses fibre strength, which could potentially improve the mechanical properties of composites [8]. According to Rosa [11], rice husk can be processed at a higher temperature as compared to wood due to its cellulose and lignin contents. Besides, with its lignocelluloses properties, it represents a potentially valuable source of fibre which could be introduced as a filler or direct substitute for wood [41]. Since rice husk usually ends up in a landfill and has no other important commercial interest, it is highly recommended to be used as reinforced fibres in composites. With the success of incorporating rice husk with reinforced composites or substitute wood in various applications, it could eventually be beneficial in terms of environmental impact, such as minimising deforestation.

Recently, natural fibres have been widely introduced as additives in additive manufacturing filaments [42]. It is also gaining popularity in various industrial sectors, such as construction, automotive, thermally insulating and sound-absorbing materials [43,44]. Engineers are always searching for new materials as well as improving processes to manufacture better products to achieve maximum efficiency, sustainability and simultaneously reduce waste. Reinforcing thermoplastic polymer with natural fibres can cause certain peculiar issues. First, the lignocellulose in natural fibre undergoes degradation during the process of constant high temperature of above 200 °C [45,46]. Therefore, the melting temperature had to be considered when choosing a suitable thermoplastic. Furthermore, natural fibre drying is important before any processing to prevent the development of water vapour during mixing, and avoid the hydrolysis of polymeric matrices [47,48]. NFRC is commonly produced through the process of continuous extrusion and used in additive manufacturing. However, this had led to several challenges such as an inhomogeneous mix between filler and polymer matrix [8], temperature control [49], as well as the creation of voids during processing [50,51]. Eventually, these result issue, including nozzle clogging in a 3D printer [49], and inconsistent mechanical performance. To improve the homogenous mixing between matrices and filler, additives such as coupling agents [8], chemical treatment [52,53] and compatibilizer [54] are added to improve the interfacial bonding between the polymer matrices and filler. Besides, the effect of processing parameters and printing parameters has not been explored deeply until recently. Table 2 shows the summarised literature investigated by various researchers in NFRC filament and their respective treatments based on some common types of thermoplastics. Each polymer, types of fibre, content percentage and presence of compatibilizer are detailed, together with their respective references. In addition, additives, such as chemical treatments [52,53,55], compatibilizer [49], plasticiser [51,56] and toughening agent [57] performed on the composites are shown. For instance, Nguyen [57] reported that toughening agent of nitrile rubber contributed considerably in terms of increased mechanical performance. Stoof [52] suggested that layer adhesion, reduce in pore size, surface finish and the mechanical performance of the composite can be improved with the addition of a plasticiser. Besides, it can be seen that the fibre amount rarely exceeded 30 wt.%. Higher fibre amount eventually results in more complex printing in the 3D printer such as a non-homogenous mixture of NFRC that could cause blockage at the nozzle [49], as well as increase the melt viscosity, which requires higher power for extrusion. Moreover, lower polymeric matrices could result in a more brittle filament as the polymer matrix that can wet the fibres decreases [57].

## 3. Processing of Natural Fibres and Fabrication of Filament

### 3.1. Pre-Processing of Fibres

Most plant-based natural fibres used for NFRCs are lignocellulosic, in which the major components of the fibres are cellulose and lignin with small amounts of other constituents, such as pectin, wax and moisture, in which the composition vary depending on the source of fibre. The main issue with these fibres is the incompatibility with the polymer matrix when compounded together [58]. The natural fibre surface has a high concentration of hydroxyl (-OH) groups which result in hydrophilicity, whereby the fibre surface attracts water. This coupled with the fact that most polymer matrices used in compounding are hydrophobic or repels water, raise an issue of compatibility between fibres and polymer matrix. The impacts of this incompatibility are significant. First, weak bonding at the fibre/polymer interface can negatively impact the mechanical properties of the composite. Fibre composites which exhibit debonding have been reported in the absence of any fibre treatment or coupling agent by Panthapulakkal et al. [58]. The debonding behaviour, which significantly reduced the mechanical properties of composite and load transfer between the matrix and fibres, is poor. Furthermore, with poor compatibility of the fibres and polymer matrix, it is harder to produce a homogenous mix. The fibres would often agglomerate and form lumps in the polymer matrix [59]. The issue of agglomeration not only affects the mechanical properties of composite, but also causes issues during the fabrication of feedstock for AM because clogging occurs very frequently. Therefore, there has been extensive research on fibre/polymer compatibility. Various methods of pre-processing to fibre were tested and proven to be effective [60,61]. Most methods were under two categories (i) modification of the fibre surface and (ii) modification of polymer matrix.

Surface treatment of fibre aims to cut down on the hydrophilicity of the fibre; hence, improve interfacial bonding between fibre and polymer matrix. This process can also make the fibre surface rougher to improve adhesion. Many methods are employed to achieve surface modification of the fibres and the methods can be further categorised as physical, chemical and mechanical surface modifications. Of the three categories, the simplest method is mechanical surface treatment [62]. The fibres are rolled or swaged to create a coarse surface to improve adhesion. These methods are usually not as popular due to the cost-effectiveness and their effect on the fibres. Therefore, most fibres pre-processing involves chemical processes.

The main purpose of a chemical process to treat the fibre is to reduce the number of hydroxyl groups on the surface, and consequently the hydrophilicity. Other processes can reduce the hydrophilicity of the fibre at one end and form chemical bonds with the polymer matrix at the other end. There are a host of chemical processes currently used to treat fibres for compounding such as silanisation, dewaxing, treatment with isocyanates and alkalisation [63]. Depending on the types of fibre and the polymers used, the chemical treatments can vary. Out of all methods listed above, the most popular surface treatment would be alkaline treatments [62]. This is primarily due to its cost-effectiveness. The main mechanism behind alkaline treatments is the removal of fibre constituents that prevents bonding with the polymer matrix, which include pectin, oils, lignin, wax and hemicellulose which is weaker than cellulose. After the removal of these constituents, it is observed that the surface of fibres is rougher and with some improvements to the mechanical properties of the composite as reported by Rajendran Royan et al. [64]. However, the success and effectiveness of this treatment, depend on the concentration of the alkaline, treatment time, type of alkaline and temperature. Researchers reported different values and performances for the composite due to a change in the alkaline treatment parameters [65]. As for the physical treatment of the fibres, these involve processes such as heat treatment, gamma-ray irradiation, corona discharge, electron beam treatment and UV ozone treatment [59,66,67]. These processes can also reduce the hydroxyl groups and increase adhesion. In the case of UV/O3 treatments, there might also be an increase in active groups on the surface of the treated fibre, further promoting chemical bonding to the matrix [64].

Despite the effectiveness of surface treatment to the fibres, these methods are usually coupled with compatibilizing agents to further improve adhesion to the fibre [64]. This is the second type of pre-processing which involves the modification of polymer matrix. This process works by modifying the chemical composition of polymer matrix to promote better adhesion. One of the most popular compatibilizing agents that is widely used by many researchers is maleic anhydride (MA). MA is often grated with a specific type of polymers, such as PP or HDPE and mixed with the composite. The result of this addition is an interaction between the MAPP/MAPE and the hydroxyl groups on the fibre, which in turn improves adhesion. This method is widely used due to the improvements in the performance composite. Researchers have reported significant increases in mechanical properties when using compatibilizing agents such as (MAPP/MAPE) [68].

### 3.2. Fabrication of Filament

The current method of producing NFRC filaments for the AM process is similar to that of conventional filaments. However, the composite preparation plays an important role in the mechanical properties of composite. Processes such as sieving the fibres, drying and mixing methods have a direct impact on properties and usability of the final filament. The preparation of the composites affects how RH interacts with the polymer matrix and ultimately affects how the composite handles loads. These processes such as drying and surface treatment ensure that there is good bonding between the fibre and polymer matrix. Furthermore, pre-mixing ensures a homogeneous mix, resulting in a stronger material. Therefore, this section first discusses the current approaches in producing the composites before methods of producing the filament are discussed. The first step in preparing the NFRC is to sieve the fibre powder [9]. For NFRCs to be used in AM, especially in the FDM process, the particle size of fibres has to be as small as possible to avoid clogging at the printer nozzle. Therefore, depending on the source of fibres, the powder is sometimes sieved to ensure no large particles are mixed in with the composite. This is an essential step as later it directly affects the quality of filament. Petchwattana et al. [9] reported that in a teak wood flour/PLA composite, the composites produced with 125-µm particle size were not able to print successfully due to clogging and only those composites produced with 75 µm particle size were printed without issue. The next step is to dry the materials. This is a very common step that is reported in almost all papers for preparing NFRC. The raw materials are oven dried for a set period or until the moisture content falls below a certain level [69,70,71,72]. The main reason for this is to eliminate any form of moisture in the fibres or polymer granules. The presence of moisture in the materials could negatively impact the downstream processes such as extrusion. If the mixture has high moisture content during extrusion or hot mixing, the evaporation of water might create gas bubbles in the polymer matrix and thus voids. This will not only affect the mechanical properties of composite but also its porosity. It is a well-known fact that NFRCs are susceptible to moisture and can swell or even break if the amount of moisture uptake is sufficient. Chen et al. [70] reported a significant increase in porosity of composites as the fibre loading increased. Therefore, it is important to ensure that moisture is minimum during the preparation of filament to decrease porosity.

After the polymer and fibres are well prepared, the polymer matrix and fibre are mixed. Various methods of mixing were reported amongst researchers from first dry mixing and then melt mixing to directly extruding the filament [9,73,74]. However, from the literature reviewed, most mixing procedures follow a similar pattern. The polymer granules, fibre powder and compatibilizing agent are first to dry, and then mixed either by a dry mixer or by hand before being mixed in a dual screw extruder [73,75]. When compared to single-screw extruders, twin-screw extruders perform significantly better when compounding is required. Many industrial applications of extrusion which involve mixing and compounding plastics to either pigments or fillers utilise twin-screw extruders due to their much better compounding ability [76]. Because of this, many researchers utilise a twin-screw extruder for melt mixing the composite. The extrudate is then granulated and put through the extrusion machine again to ensure a homogeneous mix. After a homogenous mix is produced, the filament is simply produced by running the composite granules through an extrusion machine and forcing the melted composite through a die of desired diameter. For FDM processes the feeder is usually a 1.75-mm or 2.85-mm diameter filament. The diameter of this filament must stay constant or without large variations as FDM printers rely on software that assumes a constant diameter of filament. Filaments that are too small or large cause under/over extrusion, respectively, thus affecting the quality of the printed specimen [31]. Despite this, there is currently limited literature on how researchers ensure dimensional consistency when extruding the filament. There has been a limited amount of current research into the area of utilising NFRC for AM processes, more specifically the FDM process. This proves that there is still much room for research exploration in this area.

## 4. Filament Production and Printing Parameters

### 4.1. Filament Production Parameters

As discussed above, there has been limited literature on the topic of NFRC filament production. However, the procedures for producing the filament do not differ from the procedures of producing NFRC composite itself. The only step which differs is the last step, whereby the composite is extruded into a thin, long and continuous filament to be used for printing. Therefore, from the current available literature, the parameters involved in the production of an NFRC filament can be critically discussed. First, there is a common theme amongst all research on NFRC in AM, the printing technology being used seems to be always FDM [9,24,73,74,75]. This is understandable as FDM is the most popular and cost-effective form of AM available currently. The method of producing an NFRC feeder for FDM is simple. Following that, from the literature, another common theme was noticed, which is the type of polymer matrix used. All reviewed literatures have reported on the use of polylactic acid (PLA) as a polymer matrix (Table 3), with successful attempts in producing NFRC filaments with PLA. This is because FDM is currently done with a limited range of polymers such as PLA, acrylonitrile butadiene (ABS) or polyethylene terephthalate glycol modified (PETG). Amongst all these polymers, PLA is one of the most common due to its low melting temperature and low shrinkage [77]. Works on NFRC with polyethylene-based polymer matrices for AM are still extremely rare since there were complications when HDPE was used for FDM processes, following that is the particle size used in the composites. Most literature reported on the use of natural fibre powder with particle size of below 100 µm to prevent clogging, as reported by Petchwattana et al. [9]. However, there have been reports of specimens produced with particles that were larger than 100 µm, which were printed without issue. Badouard et al. [75] reported on the use of flaxseed fibres, which was 1 mm in length and printed composite without issue. However, it is worth noting that although larger particle sizes were able to produce composites that printed without issue, these researchers utilised a larger printer nozzle that reduced the chance of clogging but decreased the print detail. Table 4 shows a summary of the particle size and the corresponding nozzle size which could print the composite successfully.

Next are parameters for mixing and producing the filament itself. As previously discussed, all reviewed literature employed a twin-screw extruder for mixing and extruding the final filament. This is primarily because twin-screw extruders are excellent in compounding different materials together. Almost all works reviewed followed the same procedure of running the composite mixture through the extruder twice. First, as a mixing process and granulating the extrudate for a second run to produce a filament [9,24,73,74,75,76,77]. As for the extrusion temperatures, the values vary between 165 °C and 200 °C. The temperature settings follow a simple trend of lower temperature at the intake zone and a higher temperature at the extrusion zone. This is simply because as the material moves closer to the extrusion die, the composite needs to be more fluid to be properly extruded.

### 4.2. Printing Parameters

When printing by using FDM technology various parameters directly affect the mechanical properties of the printed specimen. These are the nozzle temperature, layer height, infill, printing speed and raster angle [82]. Because of limited literature on these parameter effects on NFRC filaments, this section outlines the current parameters used and explains the effects of these parameters based on printing with conventional filaments. First, the nozzle temperature is arguably one of the most important parameters for the FDM process. A review of current works which investigated the effect of printing parameters and their effects on the mechanical properties for FDM process revealed that the nozzle temperature is directly correlated to the layer adhesion strength, and subsequently the tensile strength of material [82]. A high temperature causes the filament to be more fluid and bonds better to the previous layer as compared to a lower temperature. This is especially important when dealing with NFRC-based filaments because certain fibres have low thermal stability and can only handle temperatures of below 200 °C [13]. From the reviewed literature, it was found that researchers typically print NFRC-based filaments at a nozzle temperature, ranging between 180 °C and 210 °C [60,74,75].

**Figure 3:**
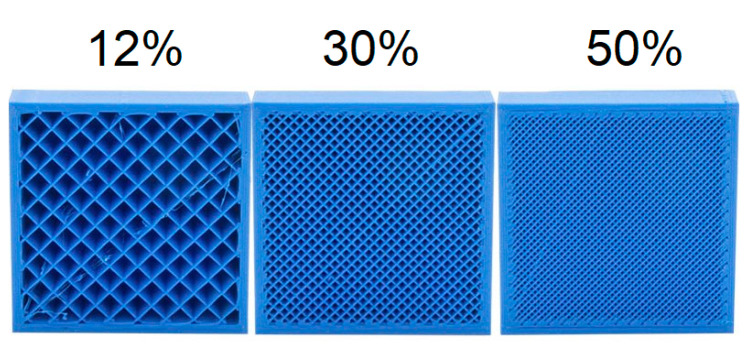
Infill percentage [84].

Layer height refers to the thickness of one layer of material deposited and is another parameter that affects the mechanical properties of a specimen. Li et al. [83] reported that the printed specimens with the smallest layer heights exhibited the best mechanical properties. Nowadays, many available FDM printers can achieve layer heights of up to 0.05 mm or 50 µm. However, these extremely low layer heights might not be practical when using NFRC filaments as the fibre particle sizes range from 75 µm to 125 µm. Therefore, current reported values for layer height when working with NFRC filaments ranged from 0.1 mm to 0.4 mm [9,73,74,75]. Infill refers to the percentage of material to empty space within a print. Shown in Figure 3 is an example of 12%, 30% and 100% infills. A lower infill percentage produces a hollower part as there is more empty space inside and vice versa. Around 12% represents the ratio of polymer to air inside the part. As the infill percentage increases, we can see a lower number of gaps in the part.

Although there are arguments in the 3D-printing community on the optimum infill percentage of being less than 100%, i.e., solid print, a study had shown that better mechanical performance was achieved as infill increased up to 100%. Alvarez et al. [85] reported that specimens printed with a 100% infill had the best impact strength amongst other specimens.

**Figure 4:**
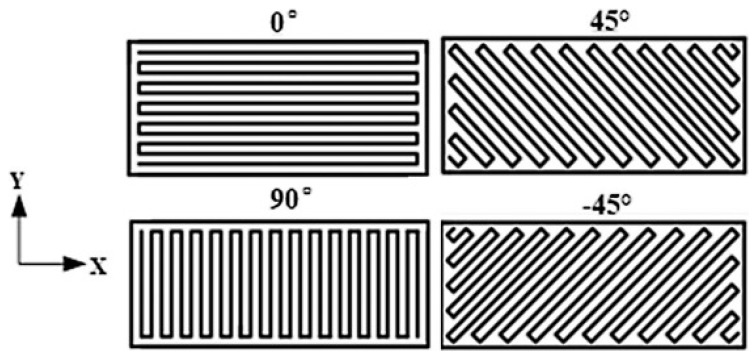
Schematic diagram of raster angle. Reprinted with permission from [86]. Copyright 2019 Elsevier.

Raster angle refers to the angle of the direction of material being deposited to the horizontal axis of machine. This angle is usually set by default at 45°. Shown in Figure 4 is an illustration of the raster angle, whereby each figure represents a cross section of a rectangular printed part. As seen in Figure 4, a 0° raster angle setting deposits material horizontally (left to right) while 90° angle deposits material vertically (up and down). The angle was measured between the direction of the printer nozzle (i.e., material deposited, and horizontal axis of printer bed plane).

Typically, the optimal condition would be to have the raster angle match the direction of loading. However, this is not always possible in practice, and thus the raster angle is always set at 45° to average out the mechanical performance. Fatimatuzahraa et al. [87] reported that a raster angle of 45° can provide superior flexural strength.

## 5. Mechanical Properties of NFRC

Concerning the mechanical properties of 3D-printed materials, the processing parameter plays a vital role in the determination of the mechanical properties. Processing parameters of a 3D printer, include the nozzle diameter, extrusion temperature, bed temperature, layer height and printing speed. The mechanical properties of the 3D-printed materials may change considerably even with one single parameter being modified [88,89]. Besides, the printing temperature is controlled between 200 °C, as most of the polymers melting temperature is lower than 200 °C. However, the temperature could not be too high since lignocelluloses in natural fibre tend to degrade at high temperatures, which could result in negative effects on the mechanical properties. On the other hand, different properties in natural fibre also influence the overall properties of composites. Therefore, the selection of natural fibre is also one of the significant factors in producing NFRC with improved mechanical properties, including tensile strength, flexural strength, impact strength, toughness, durability and hardness. Significant improvements in the mechanical properties were achieved when reinforcements of natural fibre to pure polymer were reported. Various reports of natural fibres-reinforced polymer matrix along with their resulting improved mechanical performance are tabulated in Table 3. Most papers studying the mechanical performance of bio composites filament for FDM printing dealt with PLA-based and ABS-based filaments [50,52,57,78,90]. These reported that average tensile strength ranged from 20 up to 40 MPa. With comparison between pure polymer-printed parts and reinforced ones, it is often concluded that additive of natural fibres could considerably contribute to tensile strength at specific fibre content [50,52,55,57,78,79,80,90], while stiffness increases insignificantly [50,52,55,57,79,80]. Moreover, for composites involving bio-polyethylene or polypropylene, natural fibres have improved the overall mechanical properties [55,79,80]. However, Gregor-Svetec [81] reported that the addition of cardboard dust to HDPE without any additive had resulted in inferior mechanical properties for the composites filaments. Overall, the mechanical properties of the pure polymer can be enhanced by reinforcing with natural fibres at an adequate content. A recent review article by Mazzanti et al. [8] reported that reinforcement of natural fibre to polymer increased the overall mechanical properties.

However, the mechanical properties of NFRC materials tend to be affected by the adhesion between the fibre and matrix, whereby poor adhesion could result in mechanical properties reduction [91]. Therefore, the interfacial adhesion between the natural fibre and thermoplastic matrix should be improved by modifying them. There are two types of common methods, which are the addition of coupling agents to matrix materials and surface treatments of fibre, both chemically. A coupling agent is a compound that provides a chemical bond between inorganic and organic materials [92]. In general, maleic anhydride-grafted matrices, such as MAPE used as coupling agents, had proven to improve the tensile strength of NFRC [93]. Maleic anhydride-grafted polyethylene (MAPE), or malleated polyethylene, is amongst the most popular chemical coupling agents, specifically for natural fibre polyethylene composites. It is used to compatibilize the adhesion between natural fibre and thermoplastic matrix. According to Chen et al. [69], adding the compatibilizer of MAPE had shown an increase in tensile strength and modulus of up to 35.3% and 16.6%, respectively, as compared to the NFRC without compatibilizer. Petchwattana et al. [94] reported that the overall tensile strength and flexural strength increased with the addition of MAPE between the rice husk and HDPE polymer. Therefore, it showed that using a coupling agent in natural fibre-reinforced polymer matrix composites is significant in improving their mechanical performance.

## 6. Current Optimisation Methods

The parameter settings in the 3D-printing process have a strong effect on the quality and properties of products, such as mechanical properties and dimension accuracy [95,96]. This implies the importance of optimisation of the parameter settings. To determine the optimum parameter setting, manufacturers usually have to keep on trying and reject any errors that occur. This process can be time and cost consuming. Therefore, optimisation is important in order to produce good quality 3D-printing products with shorter duration and reduced cost. Response surface methodology (RSM) is a statistical analysis tool that is used to examine the relation between several experimental variables and response variables. The basic idea of RSM is to make optimal use of a sequence of designed tests. Through the optimisation of the operating factors, RSM can be utilised to optimise the production of a specific substance. The interaction between process variables can be determined by using statistical techniques, as opposed to conventional methods. The response surface is a surface placement-based system. Therefore, the key objectives of an RSM analysis are to recognise and identify the area, whereby most suitable reaction takes place in a topography of the reaction surface, including the maximum, local, minimum and ridgelines [97]. The RSM examines the correct estimate of input and output variables and defines optimal operating conditions for the system being studied or for a factor field that meets operational requirements [98].

A Taguchi method is a descriptive analysis that identifies a product or process so that its working condition is more reliable. Taguchi method is utilised because it is easy and simple to enhance procedural factors. The technique is commonly referred to as the factorial outline of the test. In this method, exhibits called orthogonal arrays (OA) are designed extraordinarily. The methodology of orthogonal is to choose the level mix of the factors of the information plan for each trial [99]. The orthogonal array provides a slightly lower ‘deviation’ to explore a range of ways to ‘ideal setting’ the control parameters. In Taguchi, the main objective is to optimise the parameter of a process to achieve the best efficiency. If the quantity of process parameters increases, a lot of analysis must be carried out to achieve the optimised parameter. The Taguchi method uses the OA to find out the modest number of tests with the procedure parameter. The OA of Taguchi and the utilisation of ANOVA can provide the ideal combination of parameters that lead to minimal defects [100].

By selecting the proper process parameters and values, sets of a different combination by using those values are generated through design of experiment (DOE). At this stage, different types of DOE can be chosen based on the experimental requirements. The general types of DOE include Box-Behnken Design and Central Composite Design. The Box-Behnken model is an independent quadratic design because it does not have a factorial or fractional design incorporated in it. The treatment combinations of this design are at the centre and middle ends of the process field. Such designs can be rotated and require three levels per element. Compared to the central composite designs, these designs have minimal orthogonal blocking capability. The geometry of this design shows that the surface of the sphere rises over the surface of the sphere with tangential surface on each side of the space. A central composite design often requires twice the number of stars as design factors. After the selection of DOE, different combination sets of the process parameters are generated by the system. Experiments are conducted for each combination sets of data to obtain the response variable for them. Those response variables are be manually entered into the RSM system to determine the optimised parameters for the 3D-printing process. Analysis of variance (ANOVA) and contour plot are carried out after the determination of the respondent variables. ANOVA is a statistical decision-making tool that detects differences in average performance and helps test the importance of all important factors. The ANOVA method is used to understand the percentage contribution of each parameter. ANOVA is used in experimental design to decompose the total variation of the experiment into components related to the main effects and interactions, and this variation is usually called residual. The contour plot generated is used to explore the effect of a manipulated factor on the response factor. Similarly to RSM, the very first step to carry out the Taguchi method is to determine the required process parameter for the experiment and the corresponding value for that process parameter. Once the number of process parameter is obtained, the number of runs can be determined using the equation. An OA is created using Minitab with the entered process parameter and levels. After that, the responding variables are required for the OA for further analysis. Average values for the responding variables are required for the instance. After obtaining the corresponding variables for each of the process parameters and levels, it is required to find out the signal-to-noise (S/N) ratio. In experiments, the signal-to-noise ratio is valuable to identify scaling factors that are factors with a different mean and standard deviation. Scaling factors may be used without affecting signal-to-noise ratios to modify the mean on a target. The Taguchi results are then further analysed by generating the response graph to identify the impact of the control factor, whereby the parameters that affect the response variable can be identified as well. RSM is an emphatic approach—a collection, first initiated by Box and Wilson, for the modelling, problem analysing, modification and optimisation of various processes in both statistical and mathematical approaches [101]. Meanwhile, Taguchi is a special statistical method that optimises processes in search of acceptable conditions in the operating phase. This decreases the number of experiments, and thus produces the least variation [102]. In a research done by A. E. Tontowi et al., it was stated that as compared to the Taguchi method, RSM gave a better prediction on the response variable, default setting of 3D printer and dimension error. A result of the improvement in tensile strength as the response variable had been obtained, whereby the improvement by using RSM and Taguchi was 8% and 4%, respectively [103]. In another research done by Yie Hua Tan et al., the optimised results with a difference of less than 2% between RSM and Taguchi were obtained [104]. From the research, it was stated that the prediction of the optimal operating condition achieved with Taguchi was considered as a limitation as the Taguchi method requires less experimental information in the analysis and is generally utilised in linear interactions [105]. Only one optimum value was provided at a specific level. In the Taguchi method, further experiments are needed to get the future direction of optimum response. Meanwhile, RSM illustrates the statistical value of all possible interaction combinations and mathematical models, regarding 95% confidence interval and can help to identify the direction to optimum response for the future. Moreover, the RSM desirability feature can easily evaluate the optimum operating condition in the range of factors. Therefore, it is preferable and reliable to use RSM in the optimisation of the process parameters of a 3D printer to obtain a more precise optimum process parameter as compared to the Taguchi method.

## 7. Concluding Remarks

Currently, the market for natural fibres-reinforced polymers is experiencing rapid growth due to its low price, physical and mechanical and environmentally friendly properties. The production of natural fibre-reinforced polymer resolves the environmental and durability issues. One of the examples of natural fibres is rice husk. Rice husk is the product of agricultural waste. Therefore, it can be considered as a renewable resource that does not directly harm the natural resources even there is a high demand for it. Since rice husk is one of the NFRC reinforcement filler, this can be used as a replacement for the wood fibre-reinforced fibre polymer. In addition, as similar to wood fibre-reinforced fibre polymer, the rice husk-reinforced fibre polymer can also be implemented into the construction and furniture sectors. Natural fibre-reinforced polymer is more towards a wood plastic. Therefore, it can sustain from humidity and pests. With the latest developments in Europe, the high acceptance level of environmentally friendly composite materials by automobiles, government agencies and small-scale, environmentally friendly industries is expected to remain the biggest market of natural fibre-reinforced composites. With this current trend, by introducing natural fibre-reinforced polymer into additive manufacturing, the production can be further enlarged. Additive manufacturing or 3D-printing technology requires less cost as compared to subtractive manufacturing and more complex design can be achieved by using the 3D-printing technology. Therefore, improving the performance of materials would lead to the growth of composites of natural fibres in new areas.

Polymer reinforced with natural fibre could be beneficial to reduce the cost of filament yet retaining its mechanical properties. Besides, incorporating agricultural waste fibres as well as recycled polymer in the composites contributed to better sustainability.

Furthermore, it appears that natural fibre-reinforced polymer composites in general as well as additive manufacturing has shown considerable improvement in the overall mechanical properties. However, the natural fibre filling has to be at adequate content alongside with suitable additives. Interestingly, materials, such as polyethylene with semicrystalline structure have shown to be beneficial when compounded with natural fibres.

Additives such as coupling agent or compatibilizer were found to be beneficial for the mechanical properties. This is mainly because coupling agent tends to enhance the interfacial bonding of the fibre and polymer matrix.

Besides, slight drawbacks and issues result in poor processing when natural fibres are utilised. First, the material needs to be carefully dried prior to the compounding phase, as well as before printing to prevent moisture in the material from creating voids during processing. Second, the natural fibre filler should be sieved into small particle sizes (e.g., 75 µm) to eliminate the possibility of clogging at the 3D printer’s nozzle due to agglomeration. Homogenous mixing of materials is also significant to ensure consistent dispersion of materials for better and consistent mechanical characteristics of the final products.

There is a limited literature on printing with NFRC, and thus some parameters with conventional filaments were discussed. Researchers have reported using printer temperatures of 180 °C to 210 °C when printing with NFRCs. There are also reports of increased mechanical strength as layer height and infill percentage increased. Lastly, it was found that raster angles of 45° allowed the part to average out the load to produce more consistent mechanical properties.

As mentioned earlier, the parameter settings in the 3D-printing process have a strong effect on the qualities and properties of the products. Therefore, the optimisation process for the NFRC products is critical to producing high-quality products with better properties. Since there was not much optimisation research being done on the additive manufacturing on the NFRC, this may be a great gap to be fulfilled in the future.

Research towards improving the processability or flow properties should be considered as future exploration in producing natural fibre-matrix composite filament. As processing issues can have a significant detrimental impact on the final products’ mechanical properties. Mechanical, chemical and physical surface treatments (e.g., rolling, acid, alkaline, UV/O3) can improve the fibre ability to bond with the polymer matrix, producing stronger composites by modifying the surface of the fibre by different means. In addition, further research will be desirable to minimise the shrinkage and warpage drawbacks of 3D-printing HDPE composites.

## Figures and Tables

**Table 1:** Melting temperature of different thermoplastic.

Thermoplastic	Melting Temperature (°C)	Ref.
Polypropylene (PP)	150–160	[17,18,19]
Low density polyethylene (LDPE)	105–115	[20]
High-density polyethylene (HDPE)	120–190	[15,20]
Polyethylene terephthalate (PET)	235–260	[19]
Acrylonitrile butadiene styrene (ABS)	190–250	[15,21]
Polylactic Acid (PLA)	120–170	[15]

**Table 2:** Natural fibre-reinforced composites filaments.

Fibres	Polymer Matrix	Fibre Content (wt.%)	Chemical Treatment	Toughening Agent	Plasticiser	Compatibilizer	References
Hardwood lignin + carbon fibres	ABS	20–40	/	Nitrile rubber	/	/	[57]
Rice straw	ABS	0–15	/	/	/	/	[50]
Bamboo	PLA	20	/	/	PEG 600 Ester	/	[56]
Hemp	PLA	0–30	Alkaline	/	/	/	[52]
Sugarcane	PLA	3–15	Alkaline	/	/	/	[53]
Bamboo	PLA	15	/	/	cPLA1–cPLA2	/	[51]
Flax	PLA	15	/	/	cPLA1–cPLA2	/	[51]
Hemp	PP	10–30	Alkaline	/	/	MAHg-PP 2 wt.%	[55]

Note: ABS = acrylonitrile butadiene styrene; PLA = polylactide acid; PP = polypropylene.

**Table 3:** NFRC filament in AM and their resulting impact in mechanical performance.

Polymer Matrix	Fibres	Fibre Content (wt.%)	Results	Ref.
ABS	Rice straw	0–15	Overall tensile strength and modulus decreased with increased fibre content (from 35 MPa to 12 MPa); flexural stress at 30 wt.% like unfilled ABS (50 MPa)	[50]
	Lignin	0–40	Overall tensile strength increased at 40 wt.%, with nitrile rubber as additive.	[57]
PLA	Hemp	0–30	Highest tensile strength at 10 wt.% filler (37 MPa), reduced as fibre content increased; Young’s modulus from 2.5 GPa to 3 GPa	[52]
	Harakeke	0–30	Highest tensile strength at 20 wt.% filler (36.8 MPa) Young’s modulus from 2.5 GPa to 4.2 GPa	[52]
	Wood	40	Tensile strength reduced from 30 MPa to 10 MPaFlexural stress from 80 MPa to 30 MPa	[78]
PP	Hemp Harakeke	10–30	Tensile strength at 30 wt.% filler improved up to 51% as compared to unfilled PP	[55]
	Hemp Harakeke	0–30	Tensile strength at 30 wt.% filler averagely improved up to 72%; Young’s modulus at 30 wt.% filler averagely improved up to 200%. As compared to unfilled PP	[79]
bioPE	TMP	10–20	Tensile strength increased averagely from 10 Mpa (unfilled) to 29 MPa (20 wt.% filler)Stiffness increased with increase in fibre content too.	[80]
HDPE	Cardboard dust	20, 50, 70	Tensile, bending and compression strength decreased as compared to pure HDPE, mainly due to non-compatibility of particulates and pure bonding ability resulted in inferior mechanical properties.	[81]

Note: ABS = acrylonitrile butadiene styrene; PLA = polylactide acid; PP = polypropylene; PE = polyethylene; HDPE = high-density polyethylene; TMP = thermomechanical pulp.

**Table 4:** Summary of fibre particle size and nozzle size [9,24,73,74,75].

Particle Size	Nozzle Size
75 µm	0.40 mm
100 µm	0.50 mm
125 µm	0.75 mm
1 mm (length)	1.00 mm

## Data Availability

Not applicable.
